# Supplementary material for: Patterned cortical tension mediated by N-cadherin controls cell geometric order in the Drosophila eye
Source: eLife. 2017 May 24;6:e22796. doi: 10.7554/eLife.22796 (PMC5443664; doi:10.7554/eLife.22796)
Supplement: Supplementary file 1. — Table 1: MyoII levels in different experiments at different contact types. Table 2: Junction length, MyoII level, Ecad level and Ncad level in different experiments at different contact types. Table 3: Statistical value for all quantifications. Table 4: Oligos used in generating CRISPR/Cas9 mediated knock-in Ncad::mKate2 flies. DOI: http://dx.doi.org/10.7554/eLife.22796.035 [file elife-22796-supp1.docx]

**Supplementary File 1**

Table 1: MyoII levels in different experiments at different contact types

| Experiment | Type, mean±SD, n | Type, mean±SD, n | Type, mean±SD, n |
| --- | --- | --- | --- |
| MyoII levels (**zipper**) in **wildtype** ommatidium | C(E,N)\|C(E,N)  943±356  30 | P(E)\|P(E)  1687±467  22 | C(E,N)\|P(E)  2054±434  36 |
| MyoII levels (**Zipper**) in  **Ncad mutant** conditions | C(E,N)\|C(E,N)  943±356  30 | C(E)\|C(E)  1523±457  22 | C(E,N)\|C(E)  2166±538  36 |
| MyoII levels (**Sqh**) in **wildtype** ommatidium | C(E,N)\|C(E,N)  1943±404  33 | P(E)\|P(E)  2269±465  21 | C(E,N)\|P(E)  2619±498  50 |
| MyoII levels (**P-myo**) in **wildtype** ommatidium | C(E,N)\|C(E,N)  861±181  24 | P(E)\|P(E)  1018±229  19 | C(E,N)\|P(E)  1249±295  29 |
| MyoII levels **Mis-expression** of **Ncad** in Primary pigment cell | C(E,N)\|P(E, N+)  2959±465  20 | ------------------------- | C(E,N)\|P(E)  35956±354  20 |
| MyoII levels **Mis-expression** of **Ncad** in Primary pigment cell | ---------------------------- | P(E)\|P(E)  2871±364  16 | P(E)\|P(E,N+)  4248±762  16 |
| MyoII levels **Mis-expression** of  **NcadΔcyto** in Primary pigment cell | C(E,N)\|P(E,ΔN+)  2174±374  28 | ------------------------- | C(E,N)\|P(E)  2926±681  28 |
| MyoII levels **Mis-expression** of  **NcadΔcyto** in Primary pigment cell | ---------------------------- | P(E)\|P(E)  1644±331  16    P(E)\|P(E,ΔN+)  1601±315  19 | --------------------------- |

Table 2 : Junction length, MyoII level, Ecad level and Ncad level in different experiments at different contact types

| Experiment | Junction length | MyoII level | Ecad level | Ncad level |
| --- | --- | --- | --- | --- |
| Wildtype ommatidia  (single experiment) | In µm  C(E,N)\|C(E,N)  2.5±0.4  n=38    P(E)\|P(E)  3.1 ±0.3  n=18      C(E,N)\|P(E)  4.3±1.9  n=28    (Normalized to  C(E,N)\|C(E,N)=1)    1 : 1.2 : 1.7 | C(E,N)\|C(E,N)  942±355  n=30    P(E)\|P(E)  1686±467  n=22    C(E,N)\|P(E)  2054±434  n=36    (Normalized to  C(E,N)\|C(E,N)=1)    1 : 1.8 : 2.2 | C(E,N)\|C(E,N)  965±155  n=38    P(E)\|P(E)  2868±583  n=36    C(E,N)\|P(E)  2294±330  n=26    (Normalized to  C(E,N)\|C(E,N)=1)        1 : 3.0 : 2.4 | C(E,N)\|C(E,N)  2382±369  n=12      C(E,N)\|P(E)  77±52  n=20          (Normalized to  C(E,N)\|C(E,N)=1)        1 : 0 : 0.03 |
| Ncad mutant ommatidia  (single experiment) | In µm  C(E,N)\|C(E,N)  2.5±0.4  n=38    C(E)\|C(E)  1.58±0.3  n=36      C(E,N)\|C(E)  1.4±0.4  n=26    (Normalized to  C(E,N)\|C(E,N)=1)    1 : 0.6 : 0.5 | C(E,N)\|C(E,N)  942±355  n=30    C(E)\|C(E)  1523±456  n=22      C(E,N)\|C(E)  2165±537  n=36    (Normalized to  C(E,N)\|C(E,N)=1)  1 : 1.6 : 2.3 | C(E,N)\|C(E,N)  965±155  n=38    C(E)\|C(E)  2467±327  n=36      C(E,N)\|C(E)  1654±379  n=26    (Normalized to  C(E,N)\|C(E,N)=1)      1 : 2.6 : 1.7 | ------------------------ |
| MyoII overexpression (Sqh^EE^+)    (Collected over many experiment) | (Normalized to  C\|C=1)    C(Sqh^EE^+)\|C  0.8±0.1  n=14    C(Sqh^EE^+)\|P    0.7±0.1  n=14 | ------------------ | (Normalized to  C\|C=1)    C(Sqh^EE^+)\|C  1.06±0.3  n=14    C(Sqh^EE^+)\|P    2.6±0.9  n=14 | (Normalized to  C\|C=1)    C(Sqh^EE^+)\|C  1±0.1  n=14 |
| MyoII  Mutant  (Sqh -)    (Collected over many experiments) | (Normalized to  C\|C=1)    C(Sqh-)\|C    1.3±0.3  n=20    C(Sqh-)\|P    1.9±0.4  n=20 | --------------------- | (Normalized to  C\|C=1)    C(Sqh-)\|C    1.7±0.7  n=10    C(Sqh-)\|P    1.9±0.6  n=10 | (Normalized to  C\|C=1)    C(Sqh-)\|C    1.1±0.2  n=11 |

Table 3 : Statistics

| Figure | Test | Methods | N values | Replicates | P values |
| --- | --- | --- | --- | --- | --- |
| 2C | Mann-Whitney test with Bonferroni correction  (P≥0.5, N.S) | Box plot with median, 25^th^ percentile and 75^th^ percentile  If present,  In ‘+’ are outliers | N1=30, n2=22, n3=36 cell contacts in a retina | 3 independent experiments (2C is an independent experiment). | C(E,N)\|C(E,N), P(E)\|P(E)  3.5x10^-6^  P(E)\|P(E), C(E,N)\|P(E)  0.01  C(E,N)\|P(E),  C(E,N)\|C(E,N)  3.5x10^-10^ |
| 2F | Mann-Whitney test with Bonferroni correction  (P≥0.5, N.S) | Box plot with median, 25^th^ percentile and 75^th^ percentile  In ‘+’ are outliers | n1=30, n2=22, n3=36 cell contacts in a retina | 3 independent experiments (2F is an independent experiment). | C(E,N)\|C(E,N), C(E)\|C(E)  5.0x10^-5^  C(E)\|C(E), C(E,N)\|C(E)  1.2x10^-4^  C(E,N)\|P(E), C(E,N)\|C(E,N)  1.5x10^-10^ |
| 2J | Mann-Whitney test with Bonferroni correction  (P≥0.5, N.S) | Box plot with median, 25^th^ percentile and 75^th^ percentile  In ‘+’ are outliers | N1=14, n2=18, n3=19 for each type of contact, 4-5 ablation experiment in a retina | Experiments in 4-5 retinas  (2J is not an independent experiment)   \|  \| \| --- \| \|  \| | C(E,N)\|C(E,N), P(E)\|P(E)  0.02  P(E)\|P(E), C(E,N)\|P(E)  9.3x10^-6^  C(E,N)\|P(E), C(E,N)\|C(E,N)  4.2x10^-6^ |
| 2K | Mann-Whitney test with Bonferroni correction  (P≥0.5, N.S) | Box plot with Median, 25^th^ percentile and 75^th^ percentile  In ‘+’ are outliers | n=13 for each type of contact, 2-3 ablation experiment in a retina | Experiments in 4-5 retinas  (2K is not an independent experiment) | C(E,N)\|C(E,N), C(E)\|C(E)  4.7x10^-4^  C(E)\|C(E), C(E,N)\|C(E)  0.04  C(E,N)\|P(E), C(E,N)\|C(E,N  7.6x10^-6^ |
| 3C | Mann-Whitney test with Bonferroni correction  (P≥0.5, N.S) | Box plot with Median, 25^th^ percentile and 75^th^ percentile  In ‘+’ are outliers | n1 = 20,  n2 = 20 cell contacts in a retina | 2 independent experiments (3C is an independent experiment) | C(E,N)\|P(E),  C(E,N)\|P(E,N+)  1.5x10^-4^ |
| 3D | Mann-Whitney test with Bonferroni correction  (P≥0.5, N.S) | Box plot with Median, 25^th^ percentile and 75^th^ percentile | n1 = 16,  n2 = 16 cell contacts in a retina | 2 independent experiments (3D is an independent experiment) | P(E)\|P(E), P(E)\|P(E,N+)  6.5x10^-6^ |
| 3G | Not applicable (NA) | NA | n=10 | 2 independent experiments (3G is an independent experiment) | NA |
| 3J | NA | NA | n=13 | 3 independent experiments  (3J is an independent experiment) | NA |
| 4D | Mann-Whitney test with Bonferroni correction  (P≥0.5, N.S) | Box plot with Median, 25^th^ percentile and 75^th^ percentile  In ‘+’ are outliers | n1 = 28,  n2 = 28 cell contacts in a retina | 4D is combination of 2 experiments | C(E,N)\|P(E), C(E,N)\|P(E,ΔN+)  1x10^-4^ |
| 4E | Mann-Whitney test with Bonferroni correction  (P≥0.5, N.S) | Box plot with Median, 25^th^ percentile and 75^th^ percentile  In ‘+’ are outliers | n1 = 19,  n2 = 19 cell contacts in a retina | 4E is combination of 2 experiments | P(E)\|P(E), P(E)\|P(E,ΔN+)  1.2 |
| Fig. 1 Suppl. Fig. 1 A | Mann-Whitney test with Bonferroni correction  (P≥0.5, N.S) | Box plot with Median, 25^th^ percentile and 75^th^ percentile  In ‘+’ are outliers | n1 = 12,  n2 = 20 cell contacts in a retina | 1S1A is an independent experiment | C(E,N)\|C(E,N), C(E,N)\|P(E)  6.6x10^-6^ |
| Fig. 2 Suppl. Fig. 1 D | NA | Mean, SD | Length (n=15 ommatidia)  Ecad (n=15 ommatida)  MyoII (n= 10 ommatidia) | n>5 independent experiments | NA |
| Fig. 2 Suppl. Fig. 1 C | Mann-Whitney test with Bonferroni correction  (P≥0.5, N.S) | Box plot with Median, 25^th^ percentile and 75^th^ percentile  In ‘+’ are outliers | n1 = 33,  n2 = 21, n3= 50 cell contacts in a retina | 1S1C is an independent experiment | C(E,N)\|C(E,N), P(E)\|P(E)  0.04  P(E)\|P(E), C(E,N)\|P(E)  0.04  C(E,N)\|P(E), C(E,N)\|C(E,N)  3.5x10^-10^ |
| Fig. 2 Suppl. Fig. 1 G | NA | Mean, SD | Length and Ecad (n1 = 38,  n2 = 36, n3 = 26)  MyoII (n1=30, n2=22, n3=36) | Length and Ecad measurement from an independent experiment. MyoII measurement from an independent experiment  (same as 2F) | NA |
| Fig. 2 Suppl. Fig. 1 H | Mann-Whitney test with Bonferroni correction  (P≥0.5, N.S) | Box plot with Median, 25^th^ percentile and 75^th^ percentile  In ‘+’ outliers | n1 = 17,  n2 = 12, n3= 20 cell contacts in a retina | 1S1H is an independent experiment | C(E,N)\|C(E,N), P(E)\|P(E)  0.06  P(E)\|P(E), C(E,N)\|P(E)  0.02  C(E,N)\|P(E), C(E,N)\|C(E,N)  1.2x10^-5^ |
| Fig. 2 Suppl. Fig. 2 B | Mann-Whitney test with Bonferroni correction  (P≥0.5, N.S) | Box plot with Median, 25^th^ percentile and 75^th^ percentile  In ‘+’ are outliers | n1 = 12,  n2 = 12, n3= 12 cell contacts in a retina | \| 3 independent experiments (2S2B is an independent experiment) \| \| --- \| \|  \| \|  \| \|  \| | \| C(E,N)\|C(E,N), P(E)\|P(E)  1.1x10^-4^  P(E)\|P(E), C(E,N)\|P(E) 1.1x10^-4^  C(E,N)\|P(E), C(E,N)\|C(E,N) 1.1x10^-4^ \| \| --- \| |
| Fig. 2 Suppl. Fig. 2 C | Mann-Whitney test with Bonferroni correction  (P≥0.5, N.S) | Box plot with Median, 25^th^ percentile and 75^th^ percentile  In ‘+’ are outliers | n1 = 12,  n2 = 15, n3= 15 cell contacts in a retina | \| 3 independent experiments  (2S2C is an independent experiment) \| \| --- \| \|  \| \|  \| \|  \| \|  \| | C(E,N)\|C(E,N), C(E)\|C(E)  4.2x10^-3^  C(E)\|C(E), C(E,N)\|C(E),  0.84  C(E,N)\|P(E), C(E,N)\|C(E,N)  0.05 |
| Fig. 3 Suppl. Fig. 2 D | NA | NA | n=10 | Same data set as in 3G | NA |
| Fig. 3 Suppl. Fig. 2 F | Mann-Whitney test with Bonferroni correction  (P≥0.5, N.S) | Box plot with Median, 25^th^ percentile and 75^th^ percentile  In ‘+’ are outliers | n1=15, n2=15 | From 3S2G  and 3S2H | C\|C , C\|P  4.3x10^-3^ |
| Fig. 3 Suppl. Fig. 2 G | NA | NA | n=15 | 2 independent experiments (3S2G is an independent experiment) | NA |
| Fig. 3 Suppl. Fig. 2 H | NA | NA | n=15 | 3S2H is an independent experiment | NA |
| Fig. 3 Suppl. Fig. 3 C | Mann-Whitney test with Bonferroni correction  (P≥0.5, N.S) | Box plot with Median, 25^th^ percentile and 75^th^ percentile  In ‘+’ are outliers | n1 = 41,  n2 = 41 cell contacts in n>5 retina | \| n>5 independent experiments \| \| --- \| \|  \| | 0.01 |
| Fig. 5 Suppl. Fig. 1 C | NA | Box plot with Median, 25^th^ percentile and 75^th^ percentile  In ‘+’ are outlies | n=7 | Experiments in 7 retinas | NA |
| Fig. 5 Suppl. Fig. 2 C | NA | Box plot with Median, 25^th^ percentile and 75^th^ percentile  In ‘+’ are outliers | n1= 43, n2=9, n3=8, n4=28, n5=15 in n>5 mosaic retinas. n=1 simulation for each type | n>5 independent experiments | NA |
| Fig. 5 Suppl. Fig. 2 D | NA | Box plot with Median, 25^th^ percentile and 75^th^ percentile | n1= 43, n2=9, n3=8, n4=28, n5=15 in n>5 mosaic retinas. n=1 simulation for each type | n>5 independent experiments | NA |
| Fig. 5 Suppl. Fig. 3 B | Mann-Whitney test with Bonferroni correction  (P≥0.5, N.S) | Box plot with Median, 25^th^ percentile and 75^th^ percentile  In ‘+’ are outliers | n1 = 38,  n2 = 18, n3 = 28 cell contacts in a retina | 4 independent experiments  (5S3B is an independent experiment)   \|  \| \| --- \| \|  \| \|  \| \|  \| | C(E,N)\|C(E,N), P(E)\|P(E)  6.3x10^-9^  P(E)\|P(E), C(E,N)\|P(E)  1.2x10^-3^  C(E,N)\|P(E), C(E,N)\|C(E,N)  1.6x10^-11^ |
| Fig. 5 Suppl. Fig. 3 F | Mann-Whitney test with Bonferroni correction  (P≥0.5, N.S) | Box plot with Median, 25^th^ percentile and 75^th^ percentile | n1 = 38,  n2 = 36, n3 = 26 cell contacts in a retina | 4 independent experiments  (5S3F is an independent experiment) | C(E,N)\|C(E,N), C(E)\|C(E)  4.3x10^-13^  C(E)\|C(E), C(E,N)\|C(E)  1.7x10^-8^  C(E,N)\|P(E), C(E,N)\|C(E,N)  1.7x10^-8^ |
| Fig. 5 Suppl. Fig. 4 A | Mann-Whitney test with Bonferroni correction  (P≥0.5, N.S) | Box plot with Median, 25^th^ percentile and 75^th^ percentile  In ‘+’ are outliers | n1=10, n2=10, n3=10, n4=10 cell contacts from different retinas | n>5 independent experiments | C(E,N)\|C(E,N), C(E,N, Sqh-)\|C(E,N)  1.5x10^-3^  C(E,N)\|P(E), C(E,N, Sqh-)\|P(E)  1.6x10^-6^ |
| Fig. 5 Suppl. Fig. 4 B | Mann-Whitney test with Bonferroni correction  (P≥0.5, N.S) | Box plot with Median, 25^th^ percentile and 75^th^ percentile  In ‘+’ are outliers | n1=7, n2=7, n3=7, n4=7 cell contacts from different retinas | n>5 independent experiments | C(E,N)\|C(E,N), C(E,N, Sqh+)\|C(E,N)  4.6x10^-4^  C(E,N)\|P(E), C(E,N, Sqh+)\|P(E)  7.8x10^-5^ |
| Fig. 5 Suppl. Fig. 4 E | Mann-Whitney test with Bonferroni correction  (P≥0.5, N.S) | Box plot with Median, 25^th^ percentile and 75^th^ percentile  In ‘+’ are outliers | n1= 10, n2=10, n3=10, n4=7, n5=7, n6=10 in n>5 MyoII perturbed retinas. n=1 simulation for each type | n>5 independent experiments | NA |

Table 4. Oligonucleotides used in this study

**Primers used for cloning (5'-3')**

**Ncad5'-For** TACGACTCACTATAGGGCGAATTGGGTACCGGGCCCCCCCTCGAGGTCGCCCTGGCGC

**Ncad5'-Rev** CCTGAGCTAGCCAATTCTAGTTCGGTATTGTGGGGATTGGGCGCtTCGGGTCCATACATGTTGG

**Ncad3'-For**

GCATGGACGAGCTGTACAAGTAAGCATGCGCTGGTGGAGCGAGCA

**Ncad3'-Rev** TCACTAAAGGGAACAAAAGCTGGAGCTCCACCGCGGTGGCGGCCGCGCGACCACTGTCGATCGAC

**mKate2-For**

GAATTGGCTAGCTCAGGAGGTGGAGGCAGTATGGTGAGCGAGCTGATTAAGGAG

**mKate2-Rev**

GCTCGCTCCACCAGCGCATGCTCATCTGTGCCCCAGTTTGCTAGG

**gRNA-NCadFw**

GTCGGGCCAACATGTATGGACCCG

**gRNA-NCadRev**

AAACCGGGTCCATACATGTTGGCC

**Primers used for the screen of the injected flies**

**m-Kate2-Fw**

GGCAGTATGGTGAGCGAGCTGATTAAGGAGAACATG

**m-Kate2-Rv**

CTCATCTGTGCCCCAGTTTGCTAGGGAGG

**TIO-F**

CTCGCGATTCCAACACCTCAGCTGGAG

**TIO-R**

GCTGAGTCTCCTTCATGTGGGCAG

**Ncad-F2**

GCTCGGGCAGCACCTGTGTCAACG

**mKate2R2**

GTGGTGGTTGTTCACGGTGCCCTCCATG
